# An improved and highly sensitive microfluorimetric method for assessing susceptibility of *Plasmodium falciparum *to antimalarial drugs *in vitro*

**DOI:** 10.1186/1475-2875-5-95

**Published:** 2006-10-31

**Authors:** Neils B Quashie, Harry P de Koning, Lisa C Ranford-Cartwright

**Affiliations:** 1 Institute of Biomedical and Life Sciences, Division of Infection and Immunity, Glasgow Biomedical Research Centre, University of Glasgow, 120 University Place, Glasgow G12 8TA, Scotland, UK

## Abstract

**Background:**

The standard *in vitro *protocol currently in use for drug testing against *Plasmodium falciparum*, based on the incorporation of the purine [^3^H]-hypoxanthine, has two serious drawbacks. Firstly it is unsuitable for the testing of drugs that directly or indirectly impact on purine salvage or metabolism. Secondly, it relies on the use of expensive radiolabelled material, with added issues concerning detection, storage and waste disposal that make it unsuitable for use in many disease-endemic areas. Recently, the use of fluorochromes has been suggested as an alternative, but quenching of the fluorescence signal by the haemoglobin present in cultures of *Plasmodium falciparum*-infected erythrocytes severely limits the usefulness of this approach.

**Methods:**

In order to resolve this problem, a new PicoGreen^®^-based procedure has been developed which incorporates additional steps to remove the interfering haemoglobin. The 50% inhibitory concentration (IC_50_) values of chloroquine and pyrimethamine against *P. falciparum *laboratory lines 3D7 and K1 were determined using the new protocol.

**Results:**

The IC_50 _values of chloroquine and pyrimethamine against *P. falciparum *laboratory lines 3D7 and K1 determined with the new fluorescence-based protocol were statistically identical to those obtained using the traditional ^3^H-hypoxanthine incorporation method, and consistent with literature values.

**Conclusion:**

The new method proved to be accurate, reproducible and sensitive, and has the advantage of being non-radioactive. The improved PicoGreen^® ^method has the potential to replace traditional *in vitro *drug resistance assay techniques.

## Background

The emergence and spread of *Plasmodium falciparum *resistant to almost all available antimalarial drugs necessitates a constant monitoring of parasite susceptibility to antimalarial drugs, as well as the search for novel chemotherapeutic agents. Several *in vitro *drug sensitivity assays exist for this purpose [1-3]. *In vitro *drug sensitivity assays are generally based on culturing *P. falciparum *isolates in the presence of a range of antimalarial drug concentrations for one cycle of intraerythrocytic asexual replication or part thereof. The effect of antimalarial drugs is characterised by the inhibition of parasite growth. The most widely accepted test relies on microscopic scoring of parasitized and uninfected erythrocytes [2]. Other assessment methods involve the determination of the level of incorporation of radiolabelled precursors into parasite DNA [1,4] and colorimetric methods, which involve measurement of the levels of parasite lactate dehydrogenase (pLDH) [3,5] or histidine-rich protein II [6]. Each of these commonly-used assays has unique advantages as well as a number of known drawbacks. Parasitized cell counts using microscopes are extremely labour-intensive and time-consuming, although they are accurate. Incorporation of radiolabelled precursors requires supply, storage and disposal of radioactive material, often with a long half-life, as well as access to scintillation counters, which can be difficult in some disease-endemic areas where routine monitoring of resistance profiles is required. The major constraints in the use of the colorimetric methods are the cost and the limited supply of monoclonal antibodies (mAbs) required.

Recently, a microfluorimetric method using PicoGreen^® ^for assessing susceptibility of parasites to antiplasmodial compounds was reported [7]. PicoGreen^® ^(Invitrogen – Molecular Probes™) is a fluorochrome which selectively binds double-stranded DNA (dsDNA) by intercalation, resulting in an exceptionally high increase in fluorescence emission. PicoGreen^® ^has been shown to detect as little as 25 pg/ml of dsDNA in the presence of single stranded DNA (ssDNA), RNA, and free nucleotides [7]. Corbett and co-workers also demonstrated that replication of malaria parasites was directly proportional to the amount of PicoGreen^® ^fluorescence, with a linear relationship between parasitaemia of 0.1% and 15%.

Although the PicoGreen^® ^method was reported to be suitable for high throughput assays, the published method has an inherent problem, particularly for its use with parasite isolates collected from infected individuals with low parasitaemia in malaria-endemic areas: the possibility of a serious quenching of fluorescence resulting from the presence of haemoglobin. PicoGreen^® ^fluorescence occurs with an excitation maximum at 480 nm and an emission peak at 520 nm [7], whereas haemoglobin has a wide absorption spectrum at wavelengths between 300 and 800 nm, with a peak around 450 nm [8]. However, peak absorption wavelengths are dependent on whether haemoglobin is bound to oxygen (oxy-haemoglobin), carbon monoxide (carboxy-haemoglobin) or neither (deoxy-haemoglobin) [8], and the molecule in its degraded form (met-haemoglobin) also exhibits a different absorption spectrum [8]. Haemoglobin, therefore, has the potential to absorb most, if not all, of the fluorescence emitted by PicoGreen^®^.

A modified drug sensitivity assay using PicoGreen^® ^has been developed and tested in order to resolve the problem of fluorescence quenching. The modified method incorporates additional steps to remove contaminating haemoglobin before analysis of fluorescence. The modified method has been compared with the traditional [^3^H]-hypoxanthine accumulation method in drug sensitivity assays using chloroquine and pyrimethamine.

## Methods

### Relationship between DNA concentration and PicoGreen^® ^fluorescence

Salmon sperm DNA (Sigma-Aldrich, Poole, UK) was serially diluted with nucleic acid- and DNase-free water (ICN) to obtain a range of concentrations from 1,000 ng/ml to 1 ng/ml. One hundred and fifty microlitres of each solution were dispensed into wells of a white opaque 96-well microtitre plate (Greiner Bio-One). Fifty microlitres of lysis/fluorescence-mix, consisting of PicoGreen^® ^(1:200 in TE buffer, pH 7.5) and Triton X-100 (final concentration, 2%) in nucleic acid- and DNase-free water, were added to each well. Plates were incubated in the dark at room temperature for between 5–30 minutes and then fluorescence intensity was measured at 485 nm (excitation) and 528 nm (emission) using a Perkin Elmer LS 55 Luminescence Spectrophotometer. Control wells consisted of the highest concentration of salmon sperm DNA solution without PicoGreen^® ^or lysis/fluorescence-mix alone in nucleic acid- and DNase-free water respectively. The mean background reading from the two control wells was subtracted from the test fluorescence reading to give the final fluorescence measurement. Fluorescence (arbitrary units ranging from 1 to 1,000 units) was plotted against salmon sperm DNA concentration. The experiment was repeated in the absence of Triton X-100. All experiments were performed in triplicate for three independent experiments.

### Effect of haemoglobin on fluorescence readings

Uninfected human blood was washed in RPMI1640 medium to remove most of the white blood cells, and resuspended in medium to give samples with a haematocrit range of 1.0% to 5%. Each sample was spiked with salmon sperm DNA at a fixed concentration of 1,000 ng/ml. One hundred and fifty microlitres of each mixture were dispensed into the wells of a white opaque 96-well microtitre plate. Fifty microlitres of the lysis/fluorescence-mix were added and the fluorescence signal determined as described. Fluorescence readings were plotted against haematocrit. Control wells consisted of 150 μl blood at 1.0% haematocrit but without salmon sperm DNA, and 150 μl of 1,000 ng/ml salmon sperm DNA solution with no blood added.

### Relationship between parasitaemia and PicoGreen^® ^Fluorescence

An *in vitro *culture of parasite clone 3D7 at 2% haematocrit was diluted with uninfected erythrocytes (2% haematocrit in culture medium) to yield samples with parasitaemia ranging from 0.5% to 15%. One hundred and fifty microlitres of each sample were dispensed into each of two microtitre plates. One set of the plates was processed as described above, with direct lysis of parasitized erythrocytes by the lysis/fluorescence-mix. The other plate was centrifuged at 600 × *g *for 10 minutes to pellet the cells. The supernatant was removed and the cells re-suspended in saponin (0.15%, w/v in phosphate-buffered saline (1 × PBS)) to lyse the erythrocytes and release the malaria parasites. The plates were immediately centrifuged at 630 × *g *for 10 minutes, and the supernatant was removed. The pellet was then washed to remove all traces of haemoglobin by the addition of 200 μl of 1 × PBS to each well followed by centrifugation at 630 × *g*. The washing step was repeated twice to ensure complete removal of haemoglobin. Finally, following the removal of the supernatant, pellets were re-suspended in 150 μl of 1 × PBS. Fifty microlitres of the lysis/fluorescence-mix were added to each well of the plate and fluorescence signal determined as described above. Fluorescence reading was plotted against parasitaemia for each of the two sets of plates.

### Drug sensitivity assays with *P. falciparum*

Two culture-adapted standard laboratory lines of *P. falciparum*, 3D7 and K1, were tested for their sensitivity to chloroquine and pyrimethamine using the PicoGreen^® ^assay incorporating the saponin lysis step as described above. The same drug sensitivity assays were set up in parallel using the ^3^H-hypoxanthine incorporation method.

The two parasite lines selected were known to differ in their sensitivity to antimalarial drugs: clone 3D7 is sensitive to chloroquine and pyrimethamine [9] whilst K1 is resistant to both drugs [10]. Parasites were maintained in continuous culture using standard methods [11]. Cultures used for the drug sensitivity assay contained predominantly ring forms of the parasites; in some cases cultures were naturally synchronous, otherwise they were synchronized by brief incubation with 5% aqueous D-sorbitol using the standard method [12]. All drug sensitivity assays were performed in 96-well microtitre plates using the method described by Rieckmann and others [2,13]. Briefly, stocks of drugs were prepared initially in dimethyl sulfoxide (DMSO) and then diluted serially with culture medium (without serum) to achieve the desired concentration range. The final ranges of drug concentrations were 500 nM – 3.8 nM for chloroquine, and 2,000 nM – 15.6 nM for pyrimethamine. Each well of a microtitre plate was pre-dosed with 50 μl of the appropriate drug solution. Parasite cultures were diluted with fresh uninfected erythrocytes and RPMI1640 culture medium to achieve a starting parasitaemia of 0.5 – 1.0% and haematocrit of 2%. Two hundred microlitres of the diluted parasite culture were added to each well of the pre-dosed plates. Control wells consisted of 50 μl culture medium without drugs (but containing the same amount of DMSO as in drug wells) and 200 μl parasitized or uninfected erythrocytes. The plates were incubated at 37°C for 42–48 hours in a modular incubator (FlowLabs), gassed with a mixture of 3% CO_2_, 1% O_2_, 96% N_2_.

### Determination of IC_50 _using the microfluorimetric method

After incubation of parasites as described previously, 150 μl of culture were transferred into a white opaque 96-well microtitre plate and the plate centrifuged at 600 × *g *for 10 minutes to pellet the cells. The supernatant was removed and the cells re-suspended in saponin to lyse the erythrocytes, and the pellet washed to remove haemoglobin, as described above. Following resuspension and addition of the lysis/fluorescence mix, the plates were incubated at room temperature in the dark for between 5–30 minutes. Fluorescence intensity was measured at 485 nm (excitation) and 528 nm (emission) as described previously. Drug concentration was plotted against corresponding fluorescence reading and the 50% inhibitory concentration (IC_50_) was calculated by non-linear regression using the Prism software package (GraphPad Software, Inc.). The 50% inhibitory concentration was defined as the drug concentration that results in a fluorescence value of

0.5 × (maximum fluorescence - minimum fluorescence)     (Equation 1).

In this equation the maximum fluorescence is defined by the no drug control and the minimum fluorescence was defined by a 100% effective concentration of test drug or positive control.

### Determination of IC_50 _using the ^3^H-hypoxanthine uptake method

For each test performed using the microfluorescence method, a second plate was set up for determination of IC_50 _values by the [^3^H]-hypoxanthine incorporation method. Cultures were plated as described and incubated at 37°C for an initial period of 24 hours. 10 μl of 1.87 μM [^3^H]-hypoxanthine with a specific activity of 37 GBq/mmol (Amersham Biosciences) were then added to each well, and the plate was incubated for a further 24 hours. Cells were harvested from each well onto a fibreglass filter paper using a Filtermate Harvester (Packard Instruments). The filter paper was dried, sealed in a plastic bag and 3 ml scintillation fluid (OptiPhase HiSafe (Perkin Elmer)) added. Radioactivity incorporated into the parasites was counted with a Microbeta Wallac Trilux liquid scintillation and luminescence counter. The IC_50 _values representing 50% inhibition of uptake of [^3^H]-hypoxanthine were determined from a sigmoidal plot of log drug concentration against radiolabel incorporated into the parasites.

### Data analysis

Fifty percent inhibition concentration values were determined by nonlinear regression analysis of the plot of logarithm of concentration against fluorescence reading in the PicoGreen^® ^assay or against scintillation count (counts per minute) in the isotopic assay using Graphad PRISM version 3.02 software. Generally, the IC_50 _is defined as the concentration of antimalarial drug producing 50% inhibitory effect on parasite viability. Student's t-test was used to determine the significance of any difference in IC_50 _values obtained for each parasite/drug combination using the PicoGreen^® ^and isotopic method. Mean IC_50 _values and standard errors of the mean (s.e.m.) for each drug were determined from three independent drug sensitivity assays performed in triplicate on each plate.

## Results

### Relationship between salmon sperm DNA and fluorescence of PicoGreen^®^

**Figure 1:**
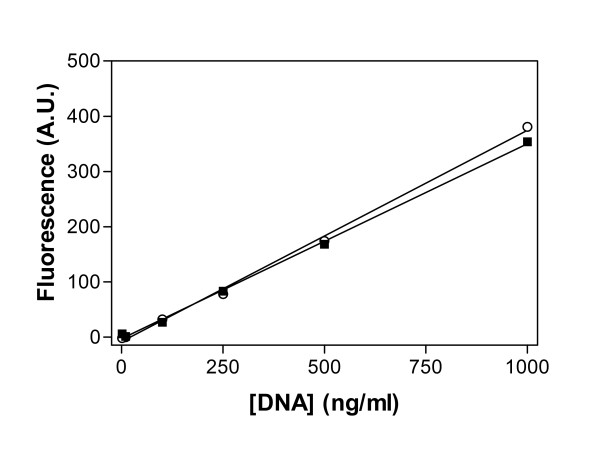
**Effect of 2% Triton X-100 on PicoGreen^® ^fluorescence with salmon sperm DNA**. PicoGreen^® ^fluorescence with salmon sperm DNA is shown in the presence (○) or absence (■) of 2% Triton X-100. Background fluorescence, defined as fluorescence detected in the absence of added DNA, was subtracted from each data point. Fluorescence is measured as arbitrary units (A.U.). Data shown are the mean of 3 independent determinations. Lines were calculated by linear regression; r^2 ^was >0.99. Analysis of the slope in the absence of Triton X-100 indicates that fluorescence increases by 3.5 ± 0.07 A.U. for every 10 ng/ml increase in DNA concentration.

A direct linear relationship was observed between PicoGreen^® ^fluorescence and salmon sperm DNA concentration (Figure 1). The presence of 2% Triton X-100 in the fluorescence-mix did not significantly change the slope of the line (P = 0.481), demonstrating that the fluorescence signal from the DNA-PicoGreen^® ^adduct was not affected by the presence of Triton X-100. The fluorescence signal was significantly reduced when the PicoGreen^® ^was dissolved in water instead of the TE buffer (data not shown). The optimal fluorescence signal was obtained with a PicoGreen^® ^dilution of 1:200 (v/v) from the stock supplied (Invitrogen).

### Effect of haemoglobin on fluorescence reading

PicoGreen^®^, in the absence of salmon sperm DNA, gave a background reading of 11.4 arbitrary fluorescence units (no DNA control). PicoGreen^® ^incubated with salmon sperm DNA at the high concentration of 1000 ng/ml did not generate a signal above background when in the presence of blood with haematocrits ranging from 1 to 5%. The control well, containing salmon sperm DNA and PicoGreen^® ^without haemoglobin, gave a fluorescence reading of around 400 units.

### Relationship between fluorescence of PicoGreen^® ^and percent parasitaemia for parasite culture

**Figure 2:**
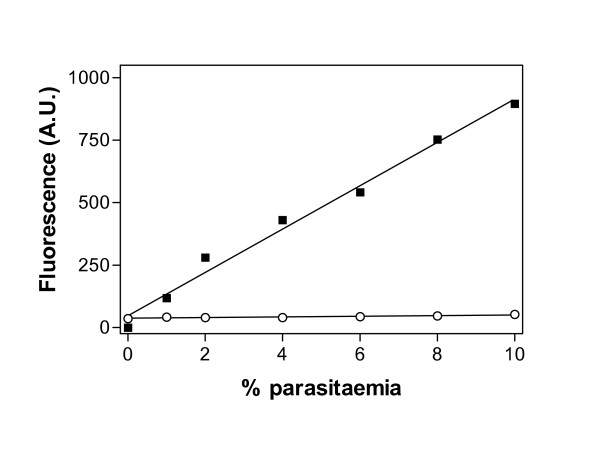
**Effect of saponin treatment on PicoGreen^® ^fluorescence**. PicoGreen^® ^fluorescence is shown following saponin treatment, followed by washing to remove haemoglobin (■), or with no saponin treatment (○), of the parasite culture (1 – 10% parasitaemia). Background fluorescence was subtracted from each point. Lines were calculated by linear regression. The slope of the line for fluorescence in the absence of saponin treatment was significantly different from zero (P = 0.005; F-test), but was only 1.5% of the slope for fluorescence after removal of haemoglobin (87 ± 4 versus 1.3 ± 0.3 A.U./% parasitaemia).

Following direct lysis of parasitized erythrocytes, only very low fluorescence of PicoGreen^® ^was detected from wells containing *P. falciparum*-infected erythrocytes with haematocrit in the range 0.5 – 5% and parasitaemia ranges up to 10% parasitaemia (Figure 2). Attempts to solubilise and precipitate the haemoglobin with a cocktail of 5 parts of quaternary ammonium hydroxide, 2 parts 30% hydrogen peroxide and 2 parts glacial acetic acid [14] could not reverse the quenching effect of this pigment (data not shown). Furthermore, varying the emission and excitation wavelength did not improve the detection of fluorescence signal.

When haemoglobin was removed through saponin-lysis and washing, PicoGreen^® ^fluorescence was restored, and a linear relationship was observed between fluorescence and percent parasitaemia (Figure 2). The mean fluorescent signal obtained following saponin treatment was significantly different from that obtained in the untreated cultures (Student's t-test, P = 0.0009).

### Comparison of IC_50 _values obtained by PicoGreen^® ^method or isotopic method

**Figure 3:**
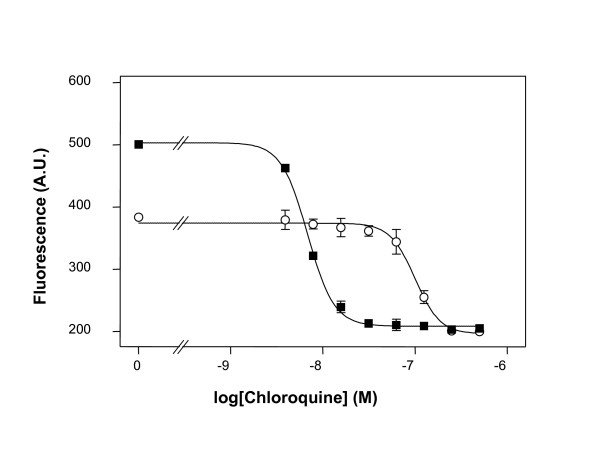
**Example of chloroquine responses of *P. falciparum *lines obtained with the improved PicoGreen^® ^method**. A typical sigmoidal graph showing the responses of *P. falciparum *lines 3D7 (■) and K1 (○) to different concentrations of chloroquine obtained with the improved PicoGreen^® ^method. Each data point is the mean of two wells and the error bars show the standard error of the mean.

**Figure 4:**
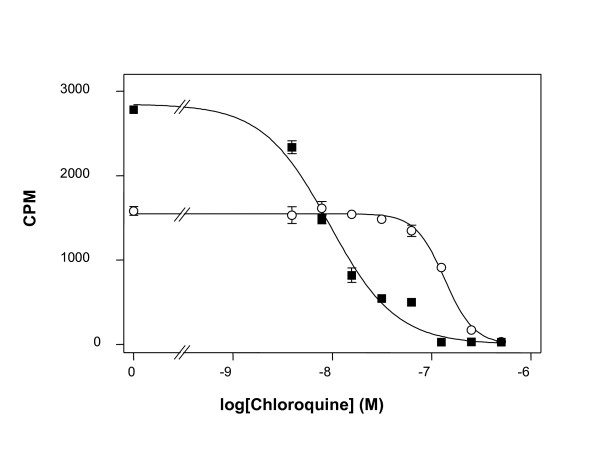
**Example of chloroquine responses of *P. falciparum *lines obtained with the traditional [^3^H]-hypoxanthine incorporation method**. A typical sigmoidal graph showing the responses of *P. falciparum *lines 3D7 (■) and K1 (○) to different concentrations of chloroquine obtained with the traditional [^3^H]-hypoxanthine incorporation method. Each data point is the mean of two wells and the error bars show the standard error of the mean.

No significant differences in IC_50 _values for chloroquine or pyrimethamine were observed between the PicoGreen^® ^method and isotopic method for either of the two parasite lines tested (Table 1). Typical graphs showing IC_50 _values for chloroquine obtained with either the standard [^3^H]-hypoxanthine accumulation assay (Figure 3) or the improved PicoGreen^® ^method (Figure 4) are presented.

**Table 1:** Mean IC_50 _values for chloroquine and pyrimethamine determined by the PicoGreen^® ^or [^3^H]-hypoxanthine accumulation assay. Mean IC_50 _values for chloroquine and pyrimethamine were determined by the PicoGreen^® ^or the [^3^H]-hypoxanthine accumulation assay for two laboratory lines of *P. falciparum*, 3D7 and K1. Data are the means of 3 independent determinations and standard error of the mean (s.e.m.) values are given in brackets. P-values were obtained from Student's t-test comparisons of IC_50 _values obtained for the two methods.

**Parasite**	**Mean IC_50 _values in nM (s.e.m.)**		
Drug	**PicoGreen**	**[^3^H]-hypoxanthine**	**P-value**
3D7			
Chloroquine	**6.3 (0.4)**	**7.2 (1.1)**	**0.47**
Pyrimethamine	**38.6 (7.6)**	**47.4 (9.6)**	**0.51**

K1			
Chloroquine	**141 (29.4)**	**128 (7.4)**	**0.69**
Pyrimethamine	**295 (115.2)**	**437 (133.4)**	**0.47**

## Discussion

Reliable and simple methods for the assessment of the sensitivity of malaria parasites to commonly-used drugs are important as public health tools, allowing evidence-based decisions on antimalarial drug policy, as well as for the evaluation of antiplasmodial activities of new chemotherapeutic compounds. Simple, reproducible assays using fluorescent labels seem to offer advantages over existing methods, especially for high throughput studies and for the routine monitoring of drug sensitivity profiles in malaria-endemic areas.

In preliminary investigations, using a previously published method [7], a linear relationship was observed between fluorescence of PicoGreen^® ^and salmon sperm DNA concentration, with a clear signal from as little as 1 ng/ml DNA in 1 × PBS. However, the addition of uninfected erythrocytes, even at low haematocrit levels, largely abolished the fluorescence seen with salmon sperm DNA. Similarly, the fluorescence signal was barely detectable from cultures of *P. falciparum*-infected erythrocytes, even at high levels of parasitaemia. It is likely that this was due to the quenching of PicoGreen^® ^fluorescence by haemoglobin, as the emission spectra for PicoGreen^® ^and the absorption spectra for haemoglobin overlap in the relevant wavelengths. Based on these observations, the published method was modified to include removal of the haemoglobin using saponin lysis of the erythrocyte and several rounds of washing with PBS. Fluorescence of the dye was thus restored and a linear relationship between fluorescence and percent parasitaemia was apparent. This linearity was reliable between 0.5 – 15% parasitaemia and was not affected by the synchronicity of the culture (data not shown).

The technique is not demanding, though caution must be exercised during the removal of the supernatant after saponin lysis or during washing with PBS, in order not to lose any of the freed parasites. The procedure lends itself well to scaling up and gives clear, accurate and indisputable results.

## Conclusion

Drug sensitivity results (IC_50_) obtained using the new method compared well with that of the traditional isotopic method for two different drugs and two distinct parasite lines. IC_50 _values were not significantly different for either assay in either parasite line tested for either chloroquine or pyrimethamine. In conclusion, the modified PicoGreen^® ^method has the potential of replacing the traditional *in vitro *drug sensitivity assays.

## Authors' contributions

NBQ performed the laboratory work and the statistical analysis of results, and helped to draft the manuscript. LRC participated in the study design and statistical analysis and drafted the manuscript. HPdeK participated in the study design and data analysis and helped to draft the manuscript. All authors read and approved the final manuscript.
